# Short-Term Cardiovascular Effects of E-Cigarettes in Adults Making a Stop-Smoking Attempt: A Randomized Controlled Trial

**DOI:** 10.3390/biology10111208

**Published:** 2021-11-19

**Authors:** Markos Klonizakis, Anil Gumber, Emma McIntosh, Leonie S. Brose

**Affiliations:** 1 Lifestyle, Exercise and Nutrition Improvement (LENI) Research Group, College of Health, Wellbeing and Life Sciences, Sheffield Hallam University, Sheffield S10 2BP, UK; A.Gumber@shu.ac.uk (A.G.); E.McIntosh@shu.ac.uk (E.M.); 2 Centre for Sport and Exercise Science, Sheffield Hallam University, Sheffield S10 2BP, UK; 3 Addictions, Institute of Psychiatry, Psychology and Neuroscience, King’s College London, London SE5 8BB, UK; leonie.brose@kcl.ac.uk; 4 SPECTRUM Research Consortium, Edinburgh EH8 9YL, UK

**Keywords:** CVD, smoking cessation, vaping, nicotine replacement therapy, vascular function

## Abstract

**Simple Summary:**

E-cigarettes are popular for smoking cessation but knowledge of their effect on cardiovascular health is limited. We compared the short-term cardiovascular effects in 248 smokers who quit smoking using e-cigarettes with or without nicotine or prescription nicotine replacement therapy (NRT). All participants received behavioural support. We assessed the cardiovascular effects of these stop smoking methods 3 days following quit date. Our work suggests that e-cigarettes offer similar vascular health benefits to that of NRT. This happens at a very early stage in the stop smoking process (3 days).

**Abstract:**

Smoking increases cardiovascular disease (CVD) risk by leading to endothelial injury. E-cigarettes remain a popular way to stop smoking. Evidence on their effect on cardiovascular health is growing but remains limited, particularly in the short-term. The main objective of this study was to compare short-term cardiovascular effects in smokers who quit smoking using e-cigarettes with or without nicotine or prescription nicotine replacement therapy (NRT). This was a single-centre (Sheffield, UK) pragmatic three-arm randomised controlled trial which recruited adult smokers (≥10 cigarettes per day), who were willing to attempt to stop smoking with support (n = 248). Participants were randomised to receive either: (a) behavioral support and e-cigarettes with 18 mg/mL nicotine (n = 84); (b) behavioral support and e-cigarettes without nicotine (n = 82); (c) behavioral support and NRT (n = 82). Flow Mediated Dilation (%FMD), peak cutaneous vascular conductance responses to acetylcholine (ACh) and sodium nitroprusside (SNP) and mean arterial pressure (MAP) were recorded at baseline and three days after stopping smoking. General Linear Models were used to compare changes between groups and changes from follow-up. Adjusting for baseline, at follow-up, all outcomes (for the 208 participants that completed the 3-day assessments) with the exception of SNP had improved significantly over baseline and there were no differences between groups (%FMD F = 1.03, p = 0.360, df = 2,207; ACh F = 0.172, p = 0.84, df = 2,207; SNP F = 0.382, p = 0.68, df = 2,207; MAP F = 0.176, p = 0.84, df = 2,207). For smokers ≥20 cigarettes per day, benefits were also pronounced. Smoking cessation showed positive cardiovascular impact even after a 3-day period and the effects did not differ between nicotine-containing e-cigarettes, nicotine-free e-cigarettes and NRT.

## 1. Introduction

Smoking increases CVD risk by leading to endothelial injury and dysfunction in both coronary and peripheral arteries [1]. It also produces an atherogenic lipid profile, primarily due by increasing triglycerides and decreasing high-density lipoprotein cholesterol [1]. Active smoking is only part of the problem, as it is now accepted that passive smoking increases heart disease death-risk by almost 30% [2].

Smoking cessation reduces the excess risk, offering cardiovascular disease risk benefits, both in the short- and in the long-term [3]. However, stopping smoking is a difficult process and pharmacological interventions, particularly using two forms of nicotine replacement therapy (NRT) or varenicline, rarely succeed on their own. This is why the combination of behavioural support and these pharmacological interventions is considered as the most effective approach [4,5,6], albeit with high relapse rates [7].

Vaping products or electronic cigarettes (e-cigarettes) have become popular as smoking cessation aids. In England, these have been the most popular choice of support for smoking cessation since 2013 and by 2018/19 were being used by about 30% of smokers making a cessation attempt [8]. Evidence from different sources indicates that they are effective for smoking cessation at least in the British context [9,10,11]. In a recent randomised controlled trial [12], e-cigarettes with behavioural support were about twice as efficacious as combination NRT with behavioural support in achieving long-term cessation (18.0% versus 9.9%, relative risk = 1.83, 95% CI: 1.30–2.58).

There is a lack of evidence on the effect of e-cigarettes on cardiovascular health. Although systematic reviews of e-cigarettes and cardiovascular effects suggest that there is potential for cardiovascular harm [13], the risk is expected to be less than that of cigarette smoking [14]. Furthermore, a recent, randomised controlled trial, which assessed cardiovascular effects of switching from smoking to e-cigarettes after one month, found significant improvements in endothelial function and vascular stiffness, two indicators for increased CVD risk [12]. Although this information is useful, additional information on the timing of improvements (particularly as of when any improvements are initiated), as well as a comparison to NRT and the use of additional cardiovascular outcome measures (e.g., to assess microvascular function) would be valuable to obtain a more comprehensive picture and help us understand how e-cigarettes physiologically affect smokers, who wish to stop smoking by using them.

Therefore, the aim of the present study was to compare short-term (3-day) cardiovascular effects in smokers who had quit smoking, using behavioural support and (a) e-cigarettes with nicotine, (b) e-cigarettes without nicotine, (c) prescription NRT. Medium- and longer-term effects will be reported separately.

## 2. Methods

The full protocol has been published elsewhere [15].

### 2.1. Design and Setting

This single-centre pragmatic three-arm randomised controlled trial was conducted in Sheffield, UK; participants were recruited between May 2017 and June 2019.

### 2.2. Participant Recruitment

Participants were recruited from the community in the wider Sheffield area [15]. Eligible were smokers who had smoked ≥10 cigarettes per day for the last year, were aged ≥18 and willing to attempt to stop smoking, using a stop smoking service or e-cigarettes. Exclusion criteria were inability to walk, recent (i.e., within 6 months) CVD events or cardiac surgery, insulin-controlled diabetes mellitus or coexisting skin conditions, leg ulceration, vasculitis or deep venous occlusion, pregnancy, major surgery scheduled during the study, contra-indications or unsuitability for NRT, current daily use of e-cigarettes and current cessation attempt supported by a stop smoking service.

### 2.3. Procedures

Following a telephone pre-screening and information about study procedures, participants were invited to the Centre for Sport and Exercise Science of Sheffield Hallam University to provide informed consent and undertake baseline assessments. Participants were enrolled by a researcher not involved in group allocation, intervention delivery or assessments. Following this visit, participants were randomised remotely into three groups by an independent statistician using a computer-generated (nQuery Advisor 6.0, Statistical Solutions, Ireland) block-randomisation stratified by gender and “pack-years” (number of packs (20 cigarettes per pack) per day times number of years smoked). The study statistician allocated a unique trial number to each participant for the study duration.

Outcome assessors were blinded to group allocation and participants were reminded regularly not to share their group allocation with assessors or those providing behavioural support. The study statistician and health economist were blinded to group allocation. Those delivering the intervention were only blinded in relation to which e-cigarette group the participants belonged to as the NRT group was receiving support through the stop smoking service. Participants were not blinded, as NRT was delivered separately, and the e-cigarette groups were able to determine the presence or absence of nicotine.

During their initial behavioural support sessions, participants set a “quit date”, on which they would stop smoking completely. This defined timing of follow-up visits.

### 2.4. Intervention

Group A and B: Both groups received behavioural support for a 3-month period. Following allocation, Groups A and B received complimentary e-cigarette equipment and refills (Tornado V5, Totally Wicked, Blackburn, UK), together with instructions on the correct usage of e-cigarettes. In both groups, participants on average received 20 bottles of 10 mL during the three-month period. Group A received nicotine-containing liquids with nicotine strength of up to 18 mg/mL. Participants could choose ice menthol or tobacco flavour (Red Label, Totally Wicked). Group B received nicotine-free liquids (0 mg/mL) with the same choice of flavours as Group A.

Group C: Following allocation, Group C participants were referred to Sheffield stop smoking services, where they received behavioural support for three months. Group C received money or shopping vouchers (depending on personal preference) as reimbursement for NRT prescription charges for the intervention period.

To ensure comparability of behavioural support provided, all groups received the same level and type of behavioural support as currently offered as standard by stop smoking services, in the form of regular face-to-face or telephone appointments as per relevant guidelines, e.g., minimum of 6 support sessions within the 3-month period [16]. All advisors had completed the same behavioural support training.

### 2.5. Measures

#### 2.5.1. Baseline

Measures included age, gender, body mass index, blood pressure, number of cigarettes and years smoked. Physical activity was measured using the SF-IPAQ [17]. Activities lasting >10 min were included, with each activity > 3 h truncated to 180 min for analysis. To arrive at a weekly figure, the metabolic equivalent (MET) value score (walking = 3.3, moderate activity = 4, vigorous activity = 8) was multiplied by the minutes the activity was carried out and again by the number of days that activity was undertaken. Additional clinical history, quality of life and CVD risk were recorded but not included in the present analysis.

The same assessor was responsible for all macro- and micro-vascular assessments. Macrovascular function was assessed using percentage change in Flow Mediated Dilation (%FMD). Smoking is associated with reduced FMD. Reduced altered brachial artery FMD is an early marker for endothelial dysfunction, a CVD risk factor [1] and can predict long-term adverse cardiovascular events [18]. FMD is a non-invasive, nitric oxide-mediated measure. Baseline scanning to assess resting vessel diameter were recorded over 3 min, following a 10-min resting period, using a Nemio XG (Toshiba, Tokyo, Japan) ultrasound machine. A 12 MHz linear transducer was used to image the brachial artery as per the International Brachial Artery Reactivity Task Force guidelines [19]. A sphygmomanometric cuff placed on the forearm was inflated at least 50 mmHg above systolic pressure to occlude artery inflow for 5 min. Recordings commenced 30 s before cuff deflation and continued for 3 min after [19]. The technical error in our lab for FMD is 5%. FMD is presented as change in post-stimulus diameter as percentage of the baseline diameter (%FMD).

Upper-body microvascular function was assessed using peak cutaneous vascular conductance (CVC) responses to acetylcholine (ACh) and sodium nitroprusside (SNP) as indicators of microvascular endothelial-dependent and -independent vasodilation, respectively, measured using Laser Doppler Fluximetry and Iontophoresis [20]. Measurements were performed in a temperature-controlled room (22–24 °C) in the ventral surface of the right arm extended to the side at heart level. Heart rate (Sports Tester, Polar, Finland) and blood pressure (left arm; Dinamap Dash 2500, GE Healthcare, Chicago, Illinois, USA) were monitored at 5-min intervals throughout the protocol.

Mean arterial pressure (MAP) was calculated using the following formula:

$$\frac{\left(2\times Diastolic\;Pressure\right)+Systolic\;Pressure}{3}$$

To obtain an index of skin blood flow, cutaneous red cell flux was measured by placing an iontophoresis laser Doppler probe (PF481-1; Perimed AB, Jarfalla, Sweden), connected to a laser Doppler fluxmeter (PF5001; Perimed AB), in the centre of each drug delivery electrode. The two drug delivery electrodes (PF383; Perimed AB) were positioned over healthy looking skin, approximately 4 cm apart with one containing 100 μL of 1% ACh (Miochol-E, Novartis, Stein, Switzerland—endothelium dependent vasodilator) and the other 100 μL of 1% SNP (Nitroprussiat, Rottapharm, Barcelona, Spain)—endothelium independent vasodilator). For additional details see Box 1. Measurements of red cell flux (recorded in arbitrary units, AU) were divided by corresponding MAP values (in mmHg) to give CVC in AU/mmHg. Here we present ACh and SNP peak CVC responses. The technical error of measurement for drug-induced, peak flux responses in our laboratory is 15% [21].

Box 1 **The iontophoresis protocol.**

**Timeline**

**Duration**
Baseline recording period4 min1st Dose (0.2 mA → 2 mC)10 s2nd Recording period4 min2nd Dose (0.2 mA → 3 mC)15 s3rd Recording period4 min3rd Dose (0.2 mA → 4 mC)20 s4th Recording period4 min4th Dose (0.3 mA → 6 mC)20 s5th Recording period4 minmA: micro-amber, mC: micro-coulomb.

#### 2.5.2. Outcomes

Outcome measures (Box 2) were recorded three days after the quit date, when all included participants reported abstinence from smoking, biochemically-validated by exhaled air measurement of <10 ppm carbon monoxide [22], with them confirming that they were following the allocated treatment. The primary outcome measure was %FMD. Secondary outcome measures were CVC max responses to ACh and SNP, and MAP (assessed as at baseline).

Box 2 **Reasons of Choice of Main Outcome Measures.**

**Outcome Measure**

**Area of Focus**

**Reasons of Choice**
Flow Mediated Dilation (%FMD)Macrovascular function (NO bioavailability at arterial level).It is a non-invasive, highly reproducible assessment. Reduced altered brachial artery FMD is considered an early marker for endothelial dysfunction, a CVD risk factor and can predict long-term adverse cardiovascular events.Cutaneous vascular conductance (CVC) responses to acetylcholine (ACh)Microvascular function (endothelial-dependent vasodilation; NO bioavailability at microvascular level).It is the only non-invasive measure that can assess reliably endothelial-dependent vasodilatory function in general and NO bioavailability in particular, at a microcirculatory level.Cutaneous vascular conductance (CVC) responses to sodium nitroprusside (SNP)Microvascular function (endothelial-independent vasodilation; smooth muscle cell function at microvascular level).It is the only non-invasive measure that can assess reliably endothelial-independent vasodilatory function. Smooth muscle cells are the most numerous components of the arterial and venous wall, playing a key role in vasodilation and in the progression of pathological conditions such as atherosclerosis.Mean arterial pressure (MAP)Macrovascular function (surrogate marker for arterial stiffness).This is a readily available, non-invasive measure (i.e., measured through Systolic and Diastolic blood pressure measurements), that can indicate changes in arterial stiffness when direct measures (e.g., pulse wave velocity) are not available or there is a need to reduce participant burden.

#### 2.5.3. Statistical Analysis

To compare changes between groups and changes from follow-up, data were analysed using General Linear Model (Univariate Analysis of Variance) with linear contrast, unadjusted and adjusted for their baseline measures with/without inclusion of covariates. For ACh and SNP, the model included BMI, age, gender, group, and years smoked. For MAP and %FMD, BMI was replaced with baseline weekly physical activity (SF-IPAQ MET).

As additional post-hoc analyses, models were run with BMI, age, gender, group, physical activity, and years smoked for a sub-sample including those smoking ≥20 cigarettes daily (n = 89 at follow-up). Although the association between exposure and cardiovascular effects is non-linear and evident even among smokers of few cigarettes, those smoking more heavily are at higher risk of cardiovascular disease [1].

Data were analysed in SPSS 24 (IBM U.K. Limited, Hampshire, UK), using an intention-to-treat approach. Missing values due to equipment malfunction were replaced by the group mean values in their respective participant’s group [23]. Statistical tests’ significance was set at p ≤ 0.05. Effect size partial η^2^ was calculated and considered “large” if >0.138, “medium” if between 0.06–0.138, and “small” if <0.06 [24].

## 3. Results

### 3.1. Participants

**Figure 1:**
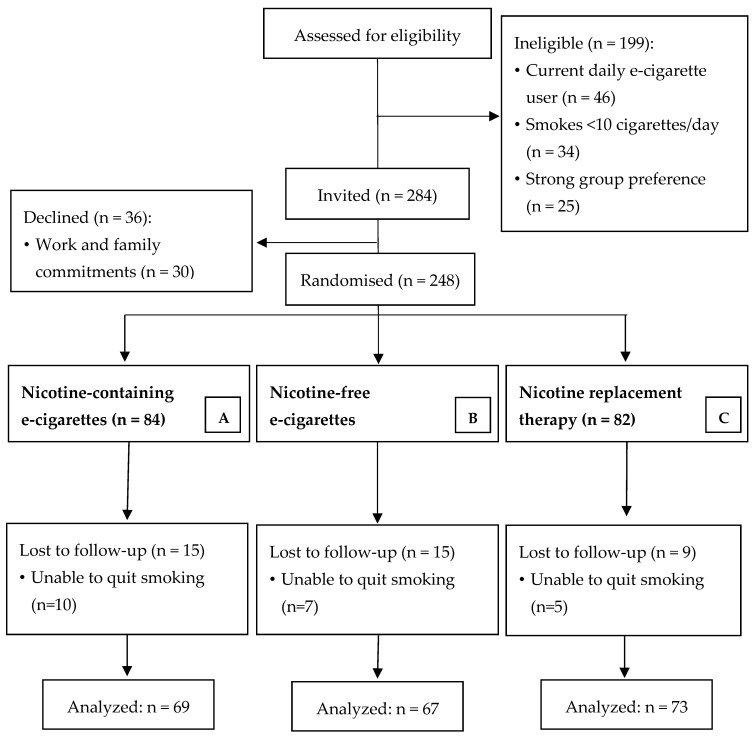
Flowchart of the Study Population.

In total, 248 participants were randomised (Figure 1). Information about their baseline characteristics is presented in Table 1. Of those, micro-vascular function was unavailable for 7 participants (Group A (nicotine-containing e-cigarettes) = 3, Group B (nicotine-free e-cigarettes) = 3, Group C (NRT) = 1). Of the randomised participants, 208 (84%) completed the follow-up visit.

### 3.2. Primary Outcome: Macrovascular Assessment

**Figure 2:**
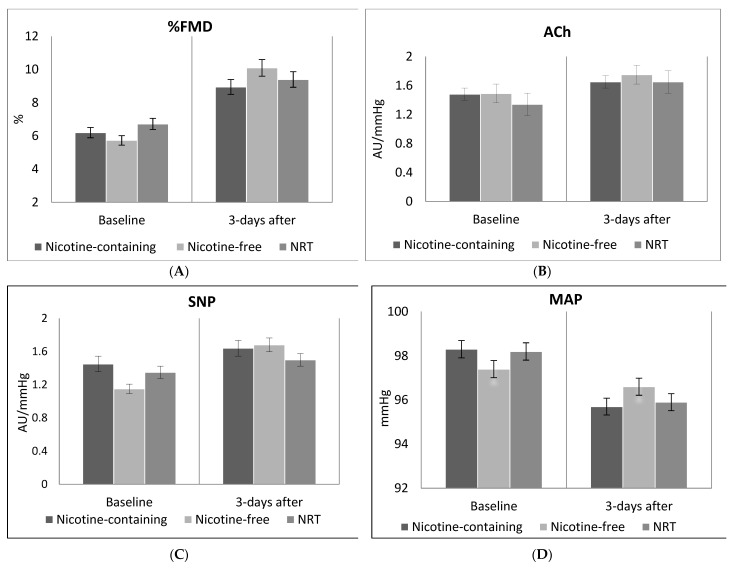
Changes from baseline to follow-up by treatment arm in (**A**): Flow mediated dilation (%FMD); (**B**): Peak cutaneous vascular conductance responses to acetylcholine (ACh); (**C**): Peak cutaneous vascular conductance responses to sodium nitroprusside (SNP); (**D**): Mean arterial pressure (MAP).

At follow-up, %FMD showed an improvement over baseline in all three groups (F = 8.99, df = 3,207; p < 0.001, with baseline %FMD: p < 0.001), with a medium effect size (η^2^ = 0.117). There was no statistically significant difference in %FMD between groups (Table 2). In analysis adjusting for group, physical activity (p < 0.02), age, gender (p < 0.02) and years smoked, the improvement in effect size from baseline to follow-up recorded (F = 5.75, df = 7,207; p < 0.001, effect size η^2^ = 0.167 ‘large’), with no significant difference between groups (Table 2; Figure 2A).

### 3.3. Secondary Outcomes: Microvascular Assessment

#### 3.3.1. Acetylcholine (ACh)

CVC values for ACh improved significantly over baseline in all three groups (F = 8.43, df = 3,207; p < 0.001, baseline ACh p < 0.001; with a medium effect size η^2^ = 0.110) and there was no difference between groups at follow-up (Table 2). The improvement from baseline was also evident with increased effect size in the models adjusting for BMI (p < 0.04), age, group, gender and years smoked (F = 4.73, df = 7,207; p < 0.001, η^2^ = 0.145 ‘large’ effect size) and there was no statistical difference between groups (Table 2; Figure 2B).

#### 3.3.2. Sodium Nitroprusside (SNP)

Although CVC values for SNP appeared to improve over the baseline, the model of adjusted mean over baseline values was not significant (F = 0.73, df = 3,207; p = 0.54, with a small effect size η^2^ = 0.011) and this remained when adjusting for BMI, age, gender and years smoked (F = 1.00, df = 3,207; p = 0.44, η^2^ = 0.034). There was no statistically-significant difference between groups (Table 2; Figure 2C).

#### 3.3.3. Mean Arterial Pressure (MAP)

MAP readings were reduced in all groups (F = 72.42, df = 3,207; p < 0.001; baseline MAP p < 0.001), with a large effect size (η^2^ = 0.516) and no difference between groups (Table 2). The reductions remained when including physical activity (p = 0.02), age, group, gender (p = 0.02) and years smoked as co-variates (F = 34.23, df = 7,207; p < 0.001, η^2^ = 0.545) with no statistically-significant difference between groups (Table 2; Figure 2D).

### 3.4. Heavy Smokers Subgroup

The post-hoc analysis for both primary (%FMD) and secondary outcome measures (ACh, SNP, MAP) showed stronger effects than in the full sample, particularly for %FMD (F = 14.53, df = 7,88; p < 0.001; η^2^ = 0.268; Table 3), for which group (p = 0.04) and physical activity (p < 0.001) were statistically significant covariates, in the fully-adjusted model. FMD improved more in the nicotine-free e-cigarette group than the nicotine-containing e-cigarette group (mean difference = 3.167 (0.590–5.744); p = 0.01). Statistical significance was reached for MAP (F = 4.24, p < 0.001, df = 7,88; η^2^ = 0.557), with gender (p = 0.02) and physical activity (p = 0.04) being statistically-significant covariates.

## 4. Discussion

In this trial, smokers who had quit three days ago showed clear improvements in different indicators of cardiovascular risk (except for SNP-assessed, endothelial-independent vasodilation). This improvement was the same for smokers using NRT, an e-cigarette with nicotine or an e-cigarette without nicotine. “Heavy smokers” (i.e., those smoking more than 20 cigarettes/day) appeared to benefit as well.

E-cigarettes are thought to increase oxidative stress levels and consequently have a negative *acute* effect on NO-bioavailability and endothelial function of healthy adults (who in theory have an “intact” endothelium) [24], although this view has recently been challenged [25]. Nevertheless, as our results indicate at both micro- and macro-vascular levels, these negative effects are less pronounced for smokers (whose endothelium is already and largely affected by smoking), who make a smoking cessation attempt using e-cigarettes or NRT. ACh is a microvascular, endothelial-dependent vasodilator, primarily associated with an increase in NO bioavailability, especially when higher doses are given, as in our study [23]. Similarly, endothelium-derived NO is considered to be a principal mediator for FMD [23], although this is questioned by some researchers [26]. As both were improved, 3-days after smoking cessation, an endothelial function improvement can be safely assumed. These findings are partially supported by the work of Carnevale et al. [24], who found the effects of e-cigarettes to be less pronounced than those caused by traditional tobacco cigarettes, especially regarding the levels of oxidative stress and NO bioavailability and to that of Haptenstall et al. [25], who found that acute e-cigarette use does not affect FMD values in smokers.

We can also accept that the observed MAP reduction suggests to some extent a reduction in arterial stiffness [27]. This is because as MAP rises, the arterial segment experiences increased circumferential pressure with greater recruitment of inelastic collagen fibres, which in turn leads to higher measured stiffness [28]. Arterial stiffness is associated with vascular damage, independently-related to adverse cardiovascular outcomes across many different patient groups and in the general population [24]. An alternative explanation for the MAP reduction in the non-nicotine study arms (i.e., NRT and nicotine-free e-cigarettes), could be the reduced sympathetic activation due to lower overall intake of sympathomimetic agents such as nicotine and particulate matter in the arms [29]. However, the fact that this reduction was a common finding among all groups suggests that the prevailing mechanism is different.

Therefore, stopping smoking through either NRT or e-cigarettes offers immediate benefits at both micro- and macro-circulatory levels. Recent work [12] reported that endothelial function and arterial stiffness in e-cigarette groups improved within 1 month of switching. The present study shows that endothelial function improves much earlier. This is an important physiological observation for the general smokers’ population, who wish to stop smoking, as the early reversal of the smoking-induced, vascular dysfunction can potentially infer a lower CVD risk [30]. However, the effects of long-term smoking abstinence while using e-cigarettes on both micro- and macro-circulatory levels remain to be investigated, although these early signs are very positive.

At this instance, we found no improvement in SNP values: SNP is an endothelial-independent vasodilator, acting through the smooth muscle cells of the micro vessels [20]. Smooth muscle cells are the most numerous components of the arterial and venous wall, playing a key role in vasodilation and in the progression of pathological conditions such as atherosclerosis [31]. Therefore, the lack of a statistically-significant improvement suggest that positive vascular effects primarily occurred in the endothelium and not in the smooth muscle (which is also affected by smoking). A longer-term observation would explore if, and at which time-point, these can be reversed.

The present work suggests that overall, in the general smokers’ population, none of the included smoking cessation methods appears to be superior over the other, in terms of short-term cardiovascular benefits, which is in line with the existing literature [12]. Pharmacological and toxicological studies support the biological plausibility that nicotine is an independent contributor to acute cardiovascular events and accelerated atherogenesis [11]. This is translated in lower FMD values (and potentially ACh-dependent microcirculatory vasodilation), experienced even after acute nicotine exposure [30]. However, nicotine is not the only constituent in smoking that impacts on cardiovascular function, e.g., CO reduces the ability to transport oxygen, acrolein increases blood cholesterol etc. Therefore, there are several elements that lead to vasoconstriction [11]. Consequently, despite the higher FMD values in the non-nicotine groups, lack of a statistically-significant difference between groups in the general smokers’ population, may suggest that the nicotine effect is one of the last to be reversed—we would need further studies, which would explore when (and if) the negative nicotine effect can be overturned.

Finally, a lower BMI and physical activity were associated with more beneficial changes which are in line with previous evidence [32,33]. This can suggest that more complex, lifestyle interventions are needed to reduce CVD risk to a greater extend.

### 4.1. Limitations

Since e-cigarettes had not been adopted as smoking cessation support option by the involved stop smoking services, there were differences in the delivery of the intervention between groups. However, as the main aim of this study is not to assess efficacy of the different options but to compare their impact on cardiovascular function, this does not affect our conclusions. Furthermore, nearly 16% of participants dropped out before the 3-day assessments—we are confident that their small number has not affected our findings.

There are many different device manufacturers as well as e-cigarette liquid producers and a wide range of liquids available so the present findings may not be generalisable to all types of devices and liquids. However, to ensure consistency and standardisation, we used a single device and liquid manufacturer throughout our study. Data on actual amount of e-liquid or NRT products used were not collected which could have provided more fine-grained information on cardiovascular effects.

Additionally, a fault in the equipment led to loss of microcirculatory assessments both at baseline and the 3-day visits for 7 participants, representing less than 3% of participants split across all three groups. Finally, we did not include a group of continuing cigarette smokers. However, all participants were smokers initially and therefore de-facto acted as controls for themselves.

### 4.2. Strengths

This is the first study to compare the short-term cardiovascular effects of smoking cessation using e-cigarettes and behavioral support with stop smoking service-delivered, NRT supported smoking cessation. Processes followed during our intervention simulated those that would be followed if e-cigarettes were widely adopted as a smoking cessation aid, in supported smoking cessation attempts in the UK and beyond. The study included an NRT-based smoking cessation group, as this represents a current common route for those wishing to stop smoking through stop smoking services or by using NRT purchased without a prescription. We explored both micro- and macro-circulation, as cardiovascular disease presents itself in both—with recent work suggesting that pathological changes in the microcirculation mirror [33] if not precede [34] changes in arteries, making it an appropriate area to study to achieve early detection.

### 4.3. Future Research

Longer-term (≥6 months) studies will shed further light as to whether CVD-risk reduction benefits persist, are increased or diminished. Moreover, additional mechanistic, large-scale studies based on blood biomarkers would allow researchers to explore mechanisms behind findings. This was not possible in the current study, due to funding limitations.

## 5. Conclusions

Smoking cessation showed positive cardiovascular impact on smokers making a stop smoking attempt, even after a short, 3-day period. The positive effects did not differ between nicotine-containing e-cigarettes, nicotine-free e-cigarettes and NRT. This suggests that in the short-term, e-cigarettes offer similar vascular health benefits to that offered by NRT, to smokers wishing to quit traditional cigarettes; thus, both e-cigarettes and NRT can be considered as CVD risk-reduction measures. This should be further explored in longer-term studies, so that stop smoking policies are scientifically informed.

## Figures and Tables

**Table 1:** Sample description, n = 248.

Baseline Characteristics	Nicotine-Containing E-Cigarettes, n = 84	Nicotine-Free E-Cigarettes, n = 82	Nicotine Replacement Therapy (NRT), n = 82
Sex, Female/Male, n	46/38	41/41	36/46
Age, Mean years (SD)	44 (14)	44 (13)	44 (13)
BMI ^a^, Mean (SD)	27.6 (5.9)	27.3 (5.4)	26.3 (5.1)
Smoking Years, Mean (SD)	24 (13)	25 (13)	25 (13)
Cigarettes per day, Mean (SD)	18 (7)	16 (7)	18 (7)
Percentage of Heavy Smokers (≥20 cigarettes per day), Mean (SD)	48%	36%	49%
Smoking packet years, Mean (SD)	23 (17)	22 (17)	24 (19)
Exhaled air carbon monoxide, Mean parts per million (SD)	15.2 (7.9)	14.3 (8.1)	16.9 (6.9)
Physical Activity, Mean weekly MET ^b^ minutes (SD)	2772 (2669)	3082 (3191)	2756 (2627)
Artery Diameter (pre-inflation), Mean (SD)	4.22 (0.66)	4.13 (0.65)	4.10 (0.56)
Artery Diameter (post-inflation), Mean (SD)	4.47 (0.64)	4.34 (0.61)	4.36 (0.55)
%FMD ^c^, Mean (SD)	6.2 (4.4)	5.6 (3.6)	6.7 (4.0)
Baseline flow (mL/min)	115.8 (74.8)	116.1 (73.5)	116.3 (73.5)
Reactive hyperaemia blood flow (mL/min)	676.8 (269.3)	678.5 (270.7)	674.5 (263.1)
Peak CVC ^d^ for Ach ^e^, Mean PU/mmHg (SD)	1.48 (1.02)	1.49 (0.98)	1.34 (1.00)
Peak CVC for SNP ^f^, Mean PU/mmHg (SD)	1.45 (1.02)	1.15 (0.72)	1.35 (0.98)
MAP ^g^, Mean mm Hg (SD)	98.3 (11.3)	97.4 (12.2)	98.2 (11.5)

^a^ BMI: Body Mass Index; ^b^ MET: Metabolic Equivalent; ^c^ %FMD: Percentage change in Flow Mediated Dilation; ^d^ CVC: Peak cutaneous vascular conductance; ^e^ ACh: Acetylcholine; ^f^ SNP: Sodium nitroprusside; ^g^ MAP: Mean Arterial Pressure.

**Table 2:** Presentation of the results for the General Linear Model for all outcomes at 3 days post quit in all participants.

3 Days Post Quit	Nicotine-Containing E-Cigarettes, n = 69	Nicotine-Free E-Cigarettes, n = 67	Nicotine Replacement Therapy (NRT), n = 73	F Value, p Value and Degrees of Freedom	η^2^
**%FMD ^a,b^**					
Unadjusted, Mean (SD)	9.0 (4.1)	10.2 (5.2)	9.4 (4.5)	F = 1.03, p = 0.360, df = 2,207	0.01
Adjusted for baseline, Mean (95% C.I.)	9.0 (7.9–10.0)	10.3 (9.2–11.4)	9.4 (8.4–10.5)	F = 8.99, p < 0.001, df = 3,207	0.117
Fully adjusted, Mean (95% C.I.)	9.0 (7.9–10)	10.3 (9.2–11.3)	9.5 (8.4–10.5)	F = 5.75, p < 0.001, df = 7,207	0.167
**Peak CVC ^c^ for ACh ^d,e^**					
Unadjusted Mean PU/mmHg (SD)	1.64 (1.06)	1.76 (1.42)	1.64 (1.18)	F = 0.172, p = 0.84, df = 2,207	0.001
Adjusted for baseline, Mean PU/mmHg (95% C.I.)	1.64 (1.38–1.91)	1.73 (1.46–2.01)	1.68 (1.42–1.95)	F = 8.43, p < 0.001, df = 3,207	0.110
Fully adjusted, Mean PU/mmHg (95% C.I.)	1.63 (1.37–1.90)	1.77 (1.49–2.05)	1.67 (1.38–1.91)	F = 4.73, p < 0.001, df = 7,207	0.145
**Peak CVC for SNP ^e,f^**					
Unadjusted Mean PU/mmHg (SD)	1.65 (1.17)	1.71 (1.61)	1.50 (1.21)	F = 0.382, p = 0.68, df = 2,207	0.004
Adjusted for baseline, Mean PU/mmHg (95% C.I.)	1.62 (1.31–1.93)	1.71 (1.39–2.03)	1.49 (1.19–1.80)	F = 0.73, p = 0.54, df = 3,207	0.011
Fully adjusted, Mean PU/mmHg (95% C.I.)	1.61 (1.30–1.92)	1.72 (1.40–2.05)	1.52 (1.21–1.83)	F = 1.00, p = 0.44, df = 7,207	0.034
**MAP ^g^**					
Unadjusted, Mean mmHg (SD)	95.7 (11.1)	96.6 (12.3)	95.9 (11.4)	F = 0.176, p = 0.84, df = 2,207	0.002
Adjusted for baseline, Mean mmHg (95% C.I.)	94.3 (93.3–97.4)	96.3 (95.4–98.6)	95.4 (95.1–98.3)	F = 72.42, p < 0.001, df = 3,207	0.516
Fully adjusted, Mean mmHg (95% C.I.)	94.3 (93.3–97.4)	96.3 (95.4–98.6)	95.4 (95.1–98.3)	F = 34.23, p < 0.001, df = 7,207	0.545

^a^ %FMD: Percentage change in Flow Mediated Dilation; ^b^ Fully adjusted models for FMD and MAP include age, gender, years smoked, physical activity (weekly MET minutes) in addition to baseline measure of outcome.; ^c^ CVC: Peak cutaneous vascular conductance; ^d^ ACh: Acetylcholine; ^e^ Fully adjusted models for ACh and SNP include BMI, age, gender, years smoked in addition to baseline measure of outcome.; ^f^ SNP: Sodium nitroprusside; ^g^ MAP: Mean Arterial Pressure.

**Table 3:** Presentation of the results for the General Linear Model for all outcomes at 3 days post quit in subgroup analysis of smokers smoking ≥20 cigarettes per day (*Heavy Smokers*).

3 Days Post Quit	Nicotine-Containing E-Cigarettes, n = 35	Nicotine-Free E-Cigarettes, n = 30	Nicotine Replacement Therapy (NRT), n = 24	F Value, p Value and Degrees of Freedom	η^2^
**%FMD ^a,b^**					
Unadjusted, Mean (SD)	8.4 (3.9)	10.7 (6.0)	10.0 (4.8)	F = 1.672, p = 0.19, df = 2,88	0.038
Adjusted for baseline, Mean (95% C.I.)	8.1 (6.4–9.8)	11.0 (9.1–13.0)	10.2 (8.6–11.8)	F = 5.53, p < 0.01, df = 3,88	0.163
Fully adjusted, Mean (95% C.I.)	8.1 (6.4–9.8)	11.2 (9.4–13.1)	10.1 (8.6–11.7)	F = 14.53, p < 0.001, df = 7,88	0.268
**Peak CVC ^c^ for ACh ^d,e^**					
Unadjusted Mean PU/mmHg (SD)	1.73 (1.17)	1.31 (0.77)	1.80 (1.25)	F = 1.371, p = 0.26, df = 2,88	0.031
Adjusted for baseline, Mean PU/mmHg (95% C.I.)	1.75 (1.37–2.12)	1.36 (0.94–1.78)	1.76 (1.41–2.11)	F = 3.98, p < 0.01, df = 3,88	0.123
Fully adjusted, Mean PU/mmHg (95% C.I.)	1.70 (1.34–2.06)	1.38 (0.94–1.82)	1.70 (1.34–2.06)	F = 2.24, p < 0.04, df = 7,88	0.168
**Peak CVC for SNP ^f,e^**					
Unadjusted Mean PU/mmHg (SD)	1.41 (0.84)	1.42 (1.34)	1.56 (1.27)	F = 0.17, p = 0.84, df = 2,88	0.004
Adjusted for baseline, Mean PU/mmHg (95% C.I.)	1.39 (0.99–1.79)	1.48 (1.02–1.93)	1.53 (1.15–1.90)	F = 1.14, p = 0.38, df = 3,88	0.039
Fully adjusted, Mean PU/mmHg (95% C.I.)	1.36 (0.95–1.78)	1.50 (1.02–1.98)	1.56 (1.20–1.98)	F = 1.04, p = 0.41, df = 7,88	0.085
**MAP ^g,b^**					
Unadjusted, Mean mmHg (SD)	99 (13)	99 (12)	97 (11)	F = 0.77, p = 0.47, df = 2,88	0.018
Adjusted for baseline, Mean mmHg (95% C.I.)	98 (95–101)	99 (96–102)	98 (95–101)	F = 29.25, p < 0.001, df = 3,88	0.508
Fully adjusted, Mean mmHg (95% C.I.)	97 (94–100)	99 (97–102)	98 (95–101)	F = 4.24, p < 0.001, df = 7,88	0.557

^a^ %FMD: Percentage change in Flow Mediated Dilation; ^b^ Fully adjusted models for FMD and MAP include age, gender, years smoked, physical activity (weekly MET minutes) in addition to baseline measure of outcome.; ^c^ CVC: Peak cutaneous vascular conductance; ^d^ ACh: Acetylcholine; ^e^ Fully adjusted models for ACh and SNP include BMI, age, gender, years smoked in addition to baseline measure of outcome.; ^f^ SNP: Sodium nitroprusside; ^g^ MAP: Mean Arterial Pressure.

## Data Availability

Restrictions apply to the availability of these data due to conditions imposed by the study’s ethical approval. Any requests should be directed to the corresponding author for consideration.
